# Unemployment and prostate cancer mortality in the OECD, 1990–2009

**DOI:** 10.3332/ecancer.2015.538

**Published:** 2015-05-14

**Authors:** Mahiben Maruthappu, Johnathan Watkins, Abigail Taylor, Callum Williams, Raghib Ali, Thomas Zeltner, Rifat Atun

**Affiliations:** 1 Faculty of Medicine, Imperial College London, London SW7 2AZ, UK; 2 Institute for Mathematical and Molecular Biomedicine, King’s College London, London SE1 1UL, UK; 3 Medical Sciences Division, University of Oxford, OX1 2JD, UK; 4 The Economist, 25 St James’s Street, London SW1A 1HG, UK; 5 Cancer Epidemiology Unit, University of Oxford, Oxford OX3 7LF, UK; 6 Faculty of Medicine and Health Sciences, United Arab Emirates University, PO Box 17666, United Arab Emirates; 7 Special Envoy for Financing to the Director General of the World Health Organization (WHO), 1211 Geneva 27, Switzerland; 8 University of Bern, Bern CH 3011, Switzerland; 9 Harvard School of Public Health, Harvard University, MA 02115, USA; † **Joint first authors**

**Keywords:** economic crisis, health economics, mortality, prostate cancer, unemployment

## Abstract

The global economic downturn has been associated with increased unemployment in many countries. Insights into the impact of unemployment on specific health conditions remain limited. We determined the association between unemployment and prostate cancer mortality in members of the Organisation for Economic Co-operation and Development (OECD). We used multivariate regression analysis to assess the association between changes in unemployment and prostate cancer mortality in OECD member states between 1990 and 2009. Country-specific differences in healthcare infrastructure, population structure, and population size were controlled for and lag analyses conducted. Several robustness checks were also performed. Time trend analyses were used to predict the number of excess deaths from prostate cancer following the 2008 global recession. Between 1990 and 2009, a 1% rise in unemployment was associated with an increase in prostate cancer mortality. Lag analysis showed a continued increase in mortality years after unemployment rises. The association between unemployment and prostate cancer mortality remained significant in robustness checks with 46 controls. Eight of the 21 OECD countries for which a time trend analysis was conducted, exhibited an estimated excess of prostate cancer deaths in at least one of 2008, 2009, or 2010, based on 2000–2007 trends. Rises in unemployment are associated with significant increases in prostate cancer mortality. Initiatives that bolster employment may help to minimise prostate cancer mortality during times of economic hardship.

## Introduction

Prostate cancer is the most commonly occurring cancer among men in many OECD countries, accounting for around one in six of all male cancer mortality in 2009 (124 deaths per 100,000 males) [1]. The reported incidence of prostate cancer is steadily increasing in almost all countries, largely because of the increased use of prostate-specific antigen (PSA) testing as an indicator [1], even though we still have relatively little understanding of its aetiology [2, 3]. Higher socioeconomic status (SES) is associated with higher incidence of prostate cancer diagnosis but with better outcomes [4, 5]. A number of studies have documented associations between lower SES and increased prostate cancer mortality, with the risk of dying increased two-fold in more deprived groups [6–8].

The global economic downturn has led to a number of OECD governments introducing economic policies that attempt to reduce budget deficits [9]. These measures, composed of tax rises and cuts in public spending, have often exacerbated already high unemployment levels [10, 11]. In Ireland, Spain, and the United States, unemployment rates in 2006 before the economic crisis were 4.4, 8.5, and 4.6, respectively. At the height of the economic crisis in 2009, these had reached 12.0, 18.0, and 9.3 respectively [12].

The economic recession has been associated with poorer health outcomes including a rise in suicide rates [11, 13, 14], increased infectious-disease incidence and mortality [10, 15–18], and decreased health-related quality of life [19]. On an individual level, unemployment, in particular, has been observed to correlate with increased mortality [20, 21]. Whilst the literature focuses on all-cause mortality as well as mental health and behaviour-related causes such as suicide, there is a lack of detailed analysis on the effect of unemployment on specific diseases. The effects of the recent economic downturn therefore raise the question of how unemployment changes, within and outside of an economic crisis, affect condition-specific health outcomes.

We sought to analyse the relationship between prostate cancer mortality and unemployment in the OECD countries between 1990 and 2009, hypothesising that increased unemployment rates would be associated with increased prostate cancer deaths because of various factors, including reduced access to health care.

## Methods

### Data collection

In an effort to reduce inter-country heterogeneity with respect to the reporting of variables such as unemployment and cancer mortality, we confined our analysis to OECD economies, which generally have agreed criteria for reporting population level variables. Data on prostate cancer mortality (age-standardised deaths per 100,000 people; ASDR) in each OECD country between 1990 and 2009 were obtained from the World Health Organisation (WHO) mortality database [22]. A prostate cancer death is identified as having the derived underlying cause code ICD-10 C61 or ICD-9 185. The quality of the data had been evaluated by the WHO [23]. Chile, Estonia, Israel, and Slovenia were excluded from the analysis because they joined the OECD after 2009. Unemployment, defined as the percentage of the labour force that is without work but available and seeking employment (World Bank data code: SL.UEM.TOTL.ZS), were obtained from the World Bank Development Indicators and Global Development Finance 2013 edition [12] (Table 1). Data used in the robustness checks were also obtained from the World Bank [12].

### Fixed-effects regression analysis

Multivariate regression analysis was used to assess the relationship between prostate cancer mortality (dependent variable) and unemployment (independent variable). To control for national variations in health care infrastructure and ensure that results were not driven by extreme observations for certain countries, a fixed-effects approach was used in the regression models, including 30 dummy variables for the 30 countries in the dataset. This allowed the models to evaluate mortality changes within individual countries while holding constant time-invariant differences (such as higher predispositions to prostate cancer as well as political, cultural, and structural differences) between countries. In effect, this conservative modelling approach made the data more comparable. To control for demographic structure, total population size and percentage of the population aged over 65 years and less than 15 years were incorporated into the model. The Cook-Weisberg test [24] was used to assess for heteroskedasticity (where sub-samples have different distributions) in the data. With the data testing positive for heteroskedasticity, robust standard errors were included in the regression models, this accounted for in part, variations in how unemployment was measured between countries. This methodology has been widely used in similar health-economic studies, and is regarded as a statistically robust and conservative approach [25–28].

Our basic linear fixed effects statistical model was as follows:

$$H_{i,t\;}-H_{i,0\;}=\alpha +(U_{i,t}-U_{i,0})\beta +\eta t+{ɛ}_{i,t},$$

where *i* is country and *t* is year; H is the health metric (prostate cancer mortality); U is the measure of unemployment; *α* represents the population structure of the country being analysed, *η* is a dummy variable for each country included in the regression model, and ɛ is the error term.

We conducted one, two, three, four, and five-year time-lag multivariate analyses to quantify the long-term effects of changes in unemployment on prostate cancer mortality. Several robustness checks were also conducted; these are detailed in the results section.

### Time trend analysis

From the original 30 OECD countries included in the study, we required countries included in our time trend analysis to have at a minimum a complete consecutive data from 2002 to 2010, inclusive. As a consequence of this inclusion criterion, we excluded Australia, Belgium, Iceland, Ireland, Italy, New Zealand, Portugal, Turkey, and Greece, leaving 21 countries. We used the years before 2007 inclusive as an observation base and fitted a linear Poisson regression model (to ensure no negative rates for decreasing trends) or a nonlinear model (for ascending trends) to these data in order to project mortality rates for the years 2008, 2009, and 2010 [29]. The geometric mean of the annual percentage change in observation-base mortality rates was used to determine whether a trend was either positive or negative. These forecasted mortality rates were then compared with the observed rates for 2008–2010 and rate ratios calculated.

Stata SE version 12 (Stata Corporation, Texas, USA) and R version 3.1.2 were used for the analysis.

## Results

**Figure 1.**
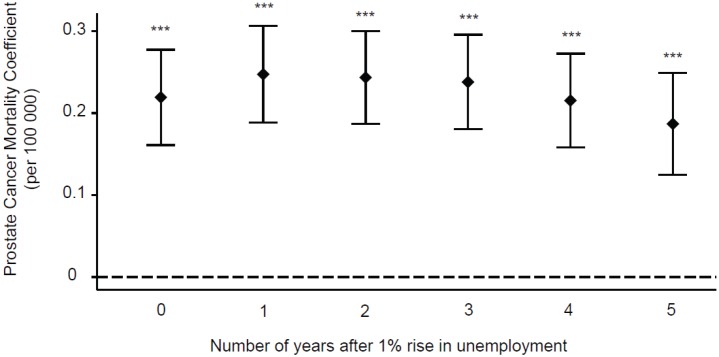
Time-lag analysis of unemployment and prostate cancer mortality. Multivariate regression analysis was used to access the relationship between prostate cancer mortality and increased unemployment. The prostate cancer mortality coefficients and their corresponding CI are displayed for the time frame of up to five years after a 1% rise in unemployment. ***p < 0.001.

Figure 1 shows the results of five regression models on OECD countries in the period 1990–2009. The results displayed are adjusted for population size, demographic structure, and variations in infrastructure. The results show that a 1% increase in unemployment is associated with a statistically significant increase in prostate cancer mortality (coefficient = 0.2193, 95% confidence interval (CI): 0.1611–0.275, *p <* 0.0001).

### Lag analysis

Further analysis was performed to investigate whether this association lasted in the longer term. The results for one, two, three, four, and five years following a 1% rise in unemployment in an OECD country during the period show that prostate cancer mortality continued to increase at one year after (coefficient = 0.2475, 95% CI: 0.1885–0.3066, *p <* 0.0001), and remained high in the subsequent years (two year coefficient = 0.2435, 95% CI: 0.1869–0.3001, *p <* 0.0001; three-year coefficient = 0.2380, 95% CI: 0.1803–0.2958, *p <* 0.0001; four year coefficient = 0.2154, 95% CI: 0.1582–0.2726, *p <* 0.0001; five year coefficient = 0.1869, 95% CI = 0.1247–0.2492, *p <* 0.0001) (Figure 1).

### Robustness checks

In order to control for confounding factors, the analysis was re-run with multiple economic, infrastructure, and health care spending controls in addition to the original controls (population size, proportion of population over 65 and under 15, and with 30 country controls). To control for economic influences, variables for changes in gross deomestic product (GDP) per capita, inflation, and interest rates were used. To control for infrastructure the effects of changes in infrastructure, controls for urbanisation, access to water, and mean calorie intake were used. Number of physicians per 100,000 and number of hospital beds per 100,000 were taken as controls of hospital resources. Finally, we re-ran the analysis controlling for out-of-pocket spending per capita. The association between a 1% rise in unemployment and increased prostate cancer mortality remained statistically significant for all individual robustness checks (Table 2), and when the analysis was run with all 46 controls simultaneously (coefficient = 0.1276, 95% CI: 0.0032–0.2519, *p =* 0.0445).

### Time trend analysis

**Figure 2.**
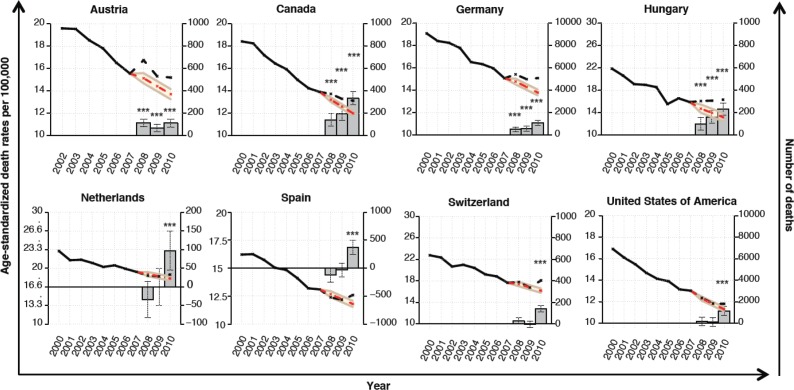
Time trend analysis of prostate cancer mortality. Time series analysis was used to assess whether actual prostate cancer mortality rates (left-hand y-axis) in 2008–2010 (black dotted line) differed from the projected rates (red dotted line) using the actual rates in 2000–2007 as an observation base (black continuous line). The estimated numbers of deaths (right-hand y-axis) resulting from this deviation (above 0) or saved (below 0) are shown as bars for 2008, 2009, and 2010. Error bars denote confidence intervals. ***p < 0.001.

To examine whether the spike in unemployment that resulted from the Great Recession of 2008 had any effect on prostate cancer mortality rates, we conducted a time trend analysis on 21 OECD countries. Among these countries, Austria, Canada, Germany, and Hungary exhibited a significantly higher prostate cancer death rate in 2008, 2009, and 2010 as compared to what would have been expected based upon rates in 2000–2007 with 2008 being the year at which the greatest deviation was observed for Austria (rate ratio = 1.1066, 95% CI: 1.0742–1.141, *p* < 0.0001; excess deaths = 114, 95% CI: 82–146), and 2010 for the other three (Canada: rate ratio = 1.095, 95% CI: 1.0765–1.1142, *p* < 0.0001; excess deaths = 333, 95% CI: 272–393; Germany: rate ratio = 1.0939, 95% CI: 1.0739–1.1147, *p* < 0.0001; excess deaths = 1,088, 95% CI: 872–1304; Hungary: rate ratio = 1.2373, 95% CI: 1.1712–1.3112, *p* < 0.0001; excess deaths = 232, 95% CI: 177–287) (Figure 2). The Netherlands, Spain, Switzerland, and the United States of America only exhibited this significantly higher deviation from the expected in 2010 (The Netherlands: rate ratio = 1.0389, 95% CI: 1.0177–1.061, *p* = 0.00035; excess deaths = 97, 95% CI: 45–149; Spain: rate ratio = 1.0675, 95% CI: 1.0435–1.0927, *p* < 0.0001; excess deaths = 372, 95% CI: 245–498; Switzerland: rate ratio = 1.1103, 95% CI: 1.0851–1.1367, *p* < 0.0001; excess deaths = 141, 95% CI: 111–171; United States: rate ratio = 1.0417, 95% CI: 1.0259–1.0579, *p* < 0.0001; excess deaths = 1142, 95% CI: 720–1564) (Figure 2). Many of the other OECD countries had trends in which the observed rates were higher than the expected rates; however, these differences were not significant. In contrast to the decreasing prostate cancer mortality rates seen in most countries, the Republic of Korea and Poland exhibited an increase and a relatively stable annual rate of prostate cancer deaths between 2000 and 2010. However, the observed mortality rates for these two countries did not differ significantly from the expected rates despite the fact that both countries experienced a sharp upturn in unemployment from 2008 onwards.

## Discussion

This study has demonstrated that increased unemployment is associated with a significant rise in prostate cancer mortality in OECD countries. This association continued for at least five years after a 1% rise in unemployment, even when controlling for economic factors, infrastructure, hospital resources, and health care spending. Because of the inclusion of such a large number of control variables, we were losing on degrees of freedom and had to reduce the sample size. Thus our results represented a highly conservative estimate of the impact of unemployment on prostate cancer mortality. Our findings were substantiated through a time trend analysis conducted on a per-country basis and examining the effect of the Great Recession of 2008 on prostate cancer mortality rates.

### Strengths and weaknesses of the study

Most commentators have studied socioeconomic inequalities, but not unemployment as a specific marker of unrest [4]. Few previous ecological studies have looked specifically at the association between changes in unemployment and cancer mortality rates. A major strength of this study is that it provides evidence for an association between a specific marker of economic crisis and a specific disease.

This study robustly demonstrated macroscopic trends in prostate cancer and unemployment in the OECD countries over a 20-year period. Data used were from a high-quality, centralised, objective database, which helped avoid selection and recall bias. The volume of data analysed allows for high statistical power and multiple robustness checks, bolstering confidence in the results obtained. Notably, our study used a conservative, fixed-effects regression analysis model. This model together with the implemented robustness checks account for many of the criticisms levelled at some of the first studies looking at the relationship between health outcomes and unemployment. Specifically, we controlled for time-invariant heterogeneity between countries; something that an aggregate time-series analyses failed to do. However, the retrospective observational study design is intrinsically prone to confoundment and bias. It is not possible to control for all possible co-dependent variables because the aetiology of prostate cancer still remains unknown. Thus, we accept that the association found in this study does not confirm causality.

It is plausible that an increase in prostate cancer for whatever reason is causing a rise in unemployment. However, given the magnitude of the unemployment increase and the relatively low unemployment rates for prostate cancer survivors compared to other cancers, this seems an unlikely explanation for our findings [30, 31]. Multiple studies have demonstrated regional and racial variation in prostate cancer mortality [32, 33]. The present study was only able to analyse trends in whole countries and in time periods of a year. Thus, we may have missed important variations at a regional intra-country level and over a shorter time-frame. The fact that we could not distinguish between different socioeconomic groups and ethnic groups is also a weakness, as we were unable to analyse whether the same groups who experience the largest increase in prostate cancer mortality are also the hardest hit by unemployment during the recession. Individual level socioeconomic data linked with prostate cancer data were not available. We were also unable to examine the extent to which access to prostate cancer screening, tumour stage at diagnosis, and aggressive treatment affected mortality in our study population, although these can be inferred from existing literature [34–36].

### Possible explanations

Although the mechanism by which unemployment may increase prostate cancer mortality is not well understood, it is likely that reduced access to prompt diagnosis and treatment in less affluent populations largely explains the discrepancy in short-term prostate cancer mortality. Reduced access to timely diagnosis may well engender a reduction in the proportion of treatable prostate cancer cases. Indeed, the related but converse effect of sharp rises in the incidence of prostate cancer–observed as a consequence of the implementation of screening programmes to detect early cases–was demonstrated in the European Randomised Study of Screening for Prostate Cancer (ERSPC) to be associated with a 21% reduction in mortality [37].

As alluded to above, stage at diagnosis is an important prognostic indicator for all tumour types, and the diagnosis of late stage prostate cancer in lower SES groups contributes to the excess mortality in this group [38, 39]. Conversely, higher SES men tend to have lower tumour grades at diagnosis, supporting the hypothesis that the more favourable outcomes of this group are because of access to screening [34]. However, the mortality gap between lower and higher SES groups persists at different Gleason scores, suggesting that baseline tumour characteristics do not wholly explain the differing survival rates [36, 40]. The remainder of the mortality gap may be explained by access to treatment, which is well-documented to be associated with wealth in the US, where low SES is an independent predictor of management by ‘watchful waiting’ rather than radiotherapy or prostatectomy [38, 41]. This is not entirely explained by the cost of private health care, as differences in quality of care for prostate cancer accessed persist even in countries with health care systems dominated by public-sector funding and service provision, such as Sweden and the UK [40, 42–44].

Although the age at diagnosis is decreasing, the mean age of prostate cancer diagnosis is 72–74 years and only 1% of cases are diagnosed younger than 50 years [45, 46]. As such, the majority of men with a prostate cancer diagnosis must have already retired, so unemployment might be expected to have relatively little impact on the outcomes from this cancer. Prostate carcinoma is found on autopsy in most men aged 85 years [47] so it is possible that the apparent increase in deaths attributed to prostate cancer may be because of an increase in all-cause mortality with prostate cancer as a comorbidity. A weakness of the present study is the inability to analyse the effect of comorbid illness on the relationship.

Racial differences in prostate cancer mortality have been well-documented [7, 48, 49], and it is difficult to separate the risks of black and minority ethnic groups from those of low SES groups [35]. These groups are also significantly more likely than white men to be unemployed [50, 51] and less likely to receive early diagnosis and aggressive treatment for prostate cancer [52]. Importantly, there is no difference in prostate cancer baseline disease characteristics or mortality between black and white men in equal-access healthcare systems in the US and the UK [53, 54], suggesting that observed racial differences in other health care systems are because of inequalities of care rather than biological factors.

Diet and body fat are thought to play a role in prostate cancer aetiology, and both are likely to change in times of economic hardship. We controlled for lack of nutrition but not for obesity, which may be a common risk factor for prostate cancer and unemployment [55–58]. Research findings on nutritional factors in prostate cancer have been inconsistent, perhaps because of the lack of differentiation between localised and aggressive subtypes of prostate cancer [59, 60]. Although lifestyle factors such as tobacco and alcohol consumption may contribute to prostate cancer mortality in association with lower SES, previous studies have found that the association is with lifetime consumption rather than current intake so these are unlikely to account for the increased mortality on the relatively short timescale studied here [61–63].

Previous studies have examined the effect of occupation on prostate cancer risk but not lack of employment. In a Dutch prospective study, only policemen had a slightly increased risk compared to the general population, and most occupations had no association with prostate cancer when controlling for potential confounding factors such as age, family history, smoking status, and diet [64]. Physical activity at work has been observed to be inversely associated with prostate cancer risk in lower SES men [65]. A study by Morris and colleagues in 1994 found that even relatively privileged people who retired early for reasons other than ill health had a significant mortality hazard compared to those who remained continuously in work. Further research on the type of work performed before unemployment may help to illuminate whether specific occupations are protective against prostate cancer.

### Implications and future directions

This study has important ramifications for clinicians, researchers, and policymakers. Clinicians need to be conscious of the higher mortality in the unemployed and be aware of the bias towards conservatively managing prostate cancer patients of lower SES [66]. Research on the comparative effectiveness of different treatment strategies on localised and advanced prostate cancer is needed to guide clinical decision-making. Public health measures to improve early detection and treatment of prostate cancer in unemployed men are needed to reduce health inequalities in OECD countries. Although meta-analyses have not confirmed the value of prostate cancer screening programmes [67], others including the ERSPC study in which prostate cancer mortality reduced by 21% in the screening group of men aged 55–69 years [37], do provide some support for such programmes. A targeted programme for unemployed and low SES men in this age group could help to reduce the burden of mortality observed in this study.

It is important for policymakers to consider the public health implications of economic austerity measures that lead to a rise in unemployment. Given that unemployment is not forecast to return to pre-recession levels in some OECD countries for several years, specific policies preventing further job losses and supporting return-to-work may improve cancer survival [68, 69]. Our work adds to the body of evidence on the problems that unemployment entails, and further work is warranted to analyse the effects of unemployment on health-related quality of life [48, 70] as well as mortality.

## Conclusions

The 2008 recession led to a rapid decline in the GDP of many member countries of the OECD, the economies of many of which have yet to recover. This event has raised the question of how macroeconomic variations may impact cancer outcomes. Our study has shown that increases in aggregate unemployment are associated with significantly worse prostate cancer mortality in OECD countries. Our study may thus be seen as a first examination of the important prostate cancer-related consequences of the economic crisis.

## List of Abbreviations

CI: confidence interval
ERSPC: European Randomised Study of Screening for Prostate Cancer
GDP: gross domestic product
ICD-10: International Classification of Diseases version 10
OECD: Organisation for Economic Co-operation and Development
PSA: prostate-specific antigen
SES: socioeconomic status
WHO: World Health Organisation

## Conflict of interest

The authors declare that they have no conflict of interest.

## Figures and Tables

**Table 1.** OECD countries, unemployment in 2009, and the average prostate cancer mortality rate between 1990–2009.

Country	Unemployment % of total labour force, 2009	Average prostate cancer mortality (ASDR per 100,000), 1990–2009
Australia	5.6	15.315
Austria	4.8	15.530
Belgium	7.9	13.225
Canada	8.3	14.055
Czech Republic	6.7	15.905
Denmark	6.0	19.235
Finland	8.2	16.665
France	9.1	14.995
Germany	7.7	14.715
Greece	9.5	9.790
Hungary	10.0	15.115
Iceland	7.2	19.360
Ireland	10.0	17.335
Italy	7.8	9.440
Japan	5.0	5.020
Republic of Korea	3.6	2.875
Luxembourg	5.1	13.825
Mexico	5.2	10.740
Netherlands	3.4	17.180
New Zealand	6.1	17.595
Norway	3.2	21.640
Poland	8.2	10.695
Portugal	9.5	12.680
Slovak Republic	12.1	15.400
Spain	18.0	12.170
Sweden	8.3	20.820
Switzerland	4.1	17.835
Turkey	14.0	–
United Kingdom	7.7	15.650
United States	9.3	13.635

ASDR, Age-standardised death rate

*Source*: World Bank Development Indicators 2013

**Table 2.** Robustness checks.

Robustness check	Controls used in multiple regression	Total number of controls in regression	Coefficient	*p* Value	Lower confidence interval	Upper confidence interval
**Economic controls**	Original analysis controls and changes in GDP per capita, inflation, interest rates	39	0.1322	0.0004	0.0595	0.2049
**Infrastructure controls**	Original analysis controls and urbanisation, access to water, nutrition (mean calorie intake)	39	0.1459	0.0000	0.0892	0.2027
**Economic and infrastructure controls**	Original analysis controls and urbanisation, access to water, nutrition (mean calorie intake), changes in GDP per capita, inflation, interest rates	42	0.0855	0.0095	0.0210	0.1499
**Hospital resource controls**	Original analysis controls and number of physicians per 100 000; number of hospital beds per 100 000	38	0.2200	0.0000	0.1474	0.2925
**Out of pocket spending control**	Original analysis controls and out of pocket spending per capita	37	0.1344	0.0002	0.0644	0.2043
**Public spending on health care control**	Original analysis controls and public spending on health care	37	0.1244	0.0004	0.0560	0.1927
**All abovementioned controls**	Original analysis controls and urbanisation, access to water, nutrition (mean calorie intake), changes in GDP per capita, inflation, interest rates, number of physicians per 100,000; number of hospital beds per 100,000; out of pocket spending, and public spending on health care	46	0.1276	0.0445	0.0032	0.2519
